# Label-Free, Single Molecule Resonant Cavity Detection: A Double-Blind Experimental Study

**DOI:** 10.3390/s150306324

**Published:** 2015-03-16

**Authors:** Maria V. Chistiakova, Ce Shi, Andrea M. Armani

**Affiliations:** Mork Family Department of Chemical Engineering and Materials Science, University of Southern California, 3651 Watt Way, Los Angeles, CA 90089, USA; E-Mails: chistiak@usc.edu (M.V.C.); cshi@usc.edu (C.S.)

**Keywords:** biosensors, optical resonators, single molecule detection

## Abstract

Optical resonant cavity sensors are gaining increasing interest as a potential diagnostic method for a range of applications, including medical prognostics and environmental monitoring. However, the majority of detection demonstrations to date have involved identifying a “known” analyte, and the more rigorous double-blind experiment, in which the experimenter must identify unknown solutions, has yet to be performed. This scenario is more representative of a real-world situation. Therefore, before these devices can truly transition, it is necessary to demonstrate this level of robustness. By combining a recently developed surface chemistry with integrated silica optical sensors, we have performed a double-blind experiment to identify four unknown solutions. The four unknown solutions represented a subset or complete set of four known solutions; as such, there were 256 possible combinations. Based on the single molecule detection signal, we correctly identified all solutions. In addition, as part of this work, we developed noise reduction algorithms.

## 1. Introduction

Over the past several decades, optical resonant cavities have demonstrated the ability to perform biological and chemical detection experiments for a wide range of applications [1,2,3,4,5,6,7,8,9,10,11,12,13,14,15,16,17,18,19,20,21,22,23,24,25,26,27,28,29,30,31,32,33,34,35,36,37]. While the initial focus was on spherical whispering gallery mode cavities, such as microdroplets [38,39] and silica microspheres [13,40], as lithography techniques became more broadly accessible, the variety of shapes and materials quickly expanded to include rings [12,41], toroids [10,42,43], photonic crystals [15,26,28,35], and many others. Additionally, as interest in pushing the detection sensitivity to the single molecule level has increased, the method of performing detection has experienced a rapid growth. While the initial demonstrations relied on detecting a refractive index change or fluorescence, more recent experiments involved detecting a change in the optical loss of the system [5], a phase change [44], back-scatter [6], or optical mode splitting [10,11]. However, the index change approach still remains the most straightforward method as it involves the minimum amount of equipment.

One reason that whispering gallery mode optical cavities are ideally suited for refractometric detection is that the optical field is confined in a circulating orbit, allowing it to sample the surface multiple times [45]. The number of orbits is directly related to the quality factor, or Q, of the cavity. It was this repeated interaction which first inspired researchers to develop these devices into sensors. Namely, the sampling results in an amplification of the signal through several distinct mechanisms: (1) every interaction acts as a unique detection event (detection is proportional to the number of circulations, which is proportional to Q); (2) the detection signal is proportional to the interaction strength of the particle and the optical field (detection is related to the intensity of the optical field at the surface); and (3) detection is performed by tracking the linewidth (detection is proportional to Q). Therefore, even a simple, intuitive analysis of this system leads one to determine that geometry and Q play critical roles in the overall sensing signals.

Given the increased interest in using these devices in diagnostics applications [1], we have performed a double-blind experiment at the single molecule level using silica optical resonant cavities. This type of experiment is standard practice in the pharmaceutical industry (also called double-blind, placebo-controlled) [46,47] and is widely recognized as the most rigorous approach to validate the reliability of a sensor’s response because it removes the experimenter’s bias. However, to our knowledge, such a study has yet to be performed with ultra-high-Q cavities at the single molecule level to date. Namely, unlike previous work in the field of resonant cavity sensing, which injected known solutions and demonstrated detection of those known analytes, we have injected unknown solutions, and successfully identified their contents based solely on the generated signal.

## 2. Experimental Section

Double-blind experiments are the classic method for verifying a diagnostic device’s ability to perform accurate identification of a chemical or biological species. In this type of experiment, a series of solutions will be prepared by one researcher (e.g., a, b, c). This series will be given to a second person who will mix and re-name the solutions (e.g., 1, 2, 3). They will also create a key that will be placed in a sealed envelope until the end of the experiment (e.g., 1 = c, 2 = a, 3 = b). The re-named set of solutions will be given to the researchers who are performing the experiment. Once the experiment is over, the researchers email both the first and second set of researchers their guess as to the identity of the solutions. At that point, the second person reveals the answers.

There are several variations on this general paradigm, depending on the complexity or difficulty desired. For example, the researchers could be told that each solution is unique. Alternatively, they could be given a long list of solutions and told that their solutions are a subset. Depending on the amount of information provided to the experimenter, the probability of arriving at the correct answer rapidly diminishes.

### 2.1. Double-Blind Strategy

In the present series of detection experiments, we are given a total of eight solutions: (1) four known solutions and (2) four unknown solutions. The four known solutions provide a ruler and are labeled with their contents: phosphate buffered saline buffer (PBS, 10 mM, pH 7.0, Sigma Aldrich), bovine serum albumin (BSA, 66 kDa, Sigma Aldrich), Streptavidin (SA, 60 kDa, Sigma Aldrich), and Streptavidin-labelled polystyrene beads (SA-PS beads, 100 nm pre-functionalization, Sigma Aldrich). The second set of four solutions is the unknown samples. All reagents (PBS) needed to create the dilutions are also supplied. It should be noted that only after the double-blind process is completed are we told the material molecular weight and manufacturer. This approach removes any bias. We are told that the unknown set of solutions represent either a subset or complete set of the known solutions. Based on this paradigm, there are 256 possible combinations. Therefore, it is highly unlikely that the correct answer would be arrived at by chance.

**Figure 1:**
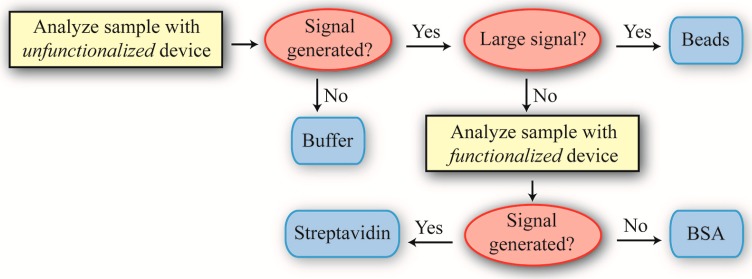
The decision tree used for differentiating between the four samples. Depending on the result from the first experiment using unfunctionalized devices, a second experiment using biotin-functionalized devices might be performed to further analyze a sample’s contents. This hierarchical approach of prioritizing decision-making is commonly used in Artificial Intelligence.

Our approach for identifying the different solutions is outlined in Figure 1. Specifically, it is straightforward to determine if a solution contained buffer or SA-PS beads. Determining if a solution contained BSA or SA required more finesse, as these molecules are approximately the same molecular weight. Therefore, we had to employ a surface functionalization capable of discriminating between BSA and SA. We chose biotin for two reasons: (1) it would improve the reliability of the SA-PS bead detection and (2) it is extremely robust, thus removing environmental instability as a potentially confounding variable.

### 2.2. Solution Preparation

All solutions are provided to the Armani Lab by an independent observer. The four known solutions (PBS buffer, BSA, SA, and SA-PS beads) are at a concentration of 1 nM. Extra PBS buffer is also given to the Armani Lab to allow dilutions of the four unknown solutions to be made. The remaining four solutions are labeled Dog, Cat, Pig, and Cow, and are also at 1 nM.

A series of solutions for detection experiments are made using serial dilution to 10 aM, 500 aM, 10 fM, and 10 pM using the provided PBS buffer. Based on previous work, we focused on the 500 aM and 1 fM solutions, and the results from the 1 fM measurements are presented here. The results from the 10 pM experiments were also used to cross-verify the results for the Cat solution. For that solution, we were concerned that the protein might have precipitated out of the PBS, resulting in an incorrect initial or starting concentration. Solutions were stored in the refrigerator until the experiments, at which point they were allowed to warm up to room temperature before testing.

### 2.3. Device Fabrication and Functionalization

**Figure 2:**
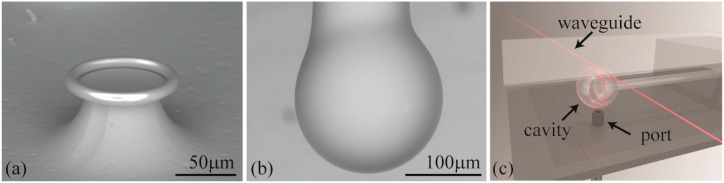
Scanning electron microscope image of a (**a**) toroid and a (**b**) microsphere resonator; (**c**) Rendering of the testing set-up with key components indicated. The liquid chamber consists of a steel holder and a glass cover slip. If a toroid is used in the experiments, then the injection port is located on the side.

Two different cavities are used in the present work: microspheres and microtoroids (Figure 2a,b). The toroidal cavities are fabricated using the standard three-step method, which is summarized as follows [48,49]. First, circular oxide pads are lithographically defined on a silicon wafer. Then, oxide microdisks are undercut using xenon difluoride. Finally, toroidal microcavities are formed using a carbon dioxide reflow process. Final device diameters ranged from 95 to 105 μm. The silica microspheres are fabricated from optical fiber by first removing the protective polymer layer from standard optical fiber. Next, it is reflowed in a carbon dioxide laser. Final device diameters ranged from 180 to 200 μm. These device diameters are chosen to minimize the radiation losses, which can occur if the device size shrinks below a critical radius [50,51,52].

To functionalize the surface of the devices with biotin, we use a previously developed NHS-Ester chemistry method [53]. In this method, the silica surface is first hydroxylated using an oxygen plasma treatment. Then, the surface is aminated using chemical vapor deposition of 3-aminopropyltrimethoxysilane. Finally, the devices are incubated in a solution of N-hydroxysuccinimido-biotin in dimethylsulfoxide. This process forms stable amide bonds and a uniform layer of biotin on the device surface. However, there are numerous other surface chemistry methods which have been developed and which could have been used to perform these experiments [54].

### 2.4. Device Characterization

Before performing a detection experiment, the quality factor of a device is determined by measuring the resonant linewidth or full-width-half-max (FWHM) of the cavity in buffer. A tunable laser (Newport, Velocity series) is coupled into a tapered optical fiber, which is brought adjacent to the resonant cavity using a nanopositioning stage. The laser is scanned across the resonance, and the spectral resolution is determined by the scan range divided by the number of data points acquired by the oscilloscope over that range. For the present series of experiments, the total wavelength scan ranges from 1 nm to approximately 0.05 nm, and the PCI card acquires 300,000 data points evenly spaced over this range. Therefore, the spectral resolution ranges from of 3.334 fm to approximately 0.166 fm. The evanescent optical field from the taper is coupled into the cavity, and the transmitted power is monitored and recorded on a NI PCI high-speed digitizer/oscilloscope with a temporal resolution of 250 MegaSamples/sec real-time.

Both the fiber and the cavity are sandwiched between the sample stage and a coverslip, and the chamber is filled with approximately 350 μL of buffer (Figure 2c). The spectra is recorded in the under-coupled regime, allowing an accurate measurement of the cavity intrinsic Q [55]. All toroid measurements are performed at 633 nm, and all microsphere measurements are performed at 765 nm. All non-functionalized cavities have intrinsic Q factors above 10 million at the start of the experiments.

### 2.5. Sensing Experiments Procedure

The coupling is increased for the detection measurements. The output power during the detection measurements is greater than 1 mW (633 nm) or 2.3 mW (765 nm). At least 90% of the laser power is coupled into the cavity. It is important to note that at these circulating powers, nonlinear phenomena such as Raman lasing [56] and opto-thermal broadening [5] have been previously observed in these devices operating under these conditions (same size device at the same wavelength in buffer).

To begin a detection measurement, the background signal is measured to establish the noise level. The minimum position and transmission error, the resonance is recorded for approximately 200 s at 10 Hz (10 points per second). After recording two noise measurements, the detection measurements are started. The LabView program begins recording the position of the peak just before the solution of interest is injected. The flow rate of each solution is set to be 50 μL/min. The solution is injected for two min, for a total injected volume of 100 μL, and then the flow is stopped. During this time, 2000 data points are recorded. Then, the liquid chamber is flushed with excess buffer, and the experiment is repeated. We chose to flow the solutions across the device surface instead of relying purely on diffusion as it this approach allows a more controlled delivery of the material to the sensing device surface [57].

The results are partially analyzed immediately after the measurement to provide instruction according to the design tree outlined in Figure 1 as to the next experiment. After the unknown solutions are tested, the known solutions are used as a reference, assuming the device is still operational; namely the Q and the circulating power are not degraded and the taper is not broken.

### 2.6. Data Analysis Methods

**Figure 3:**
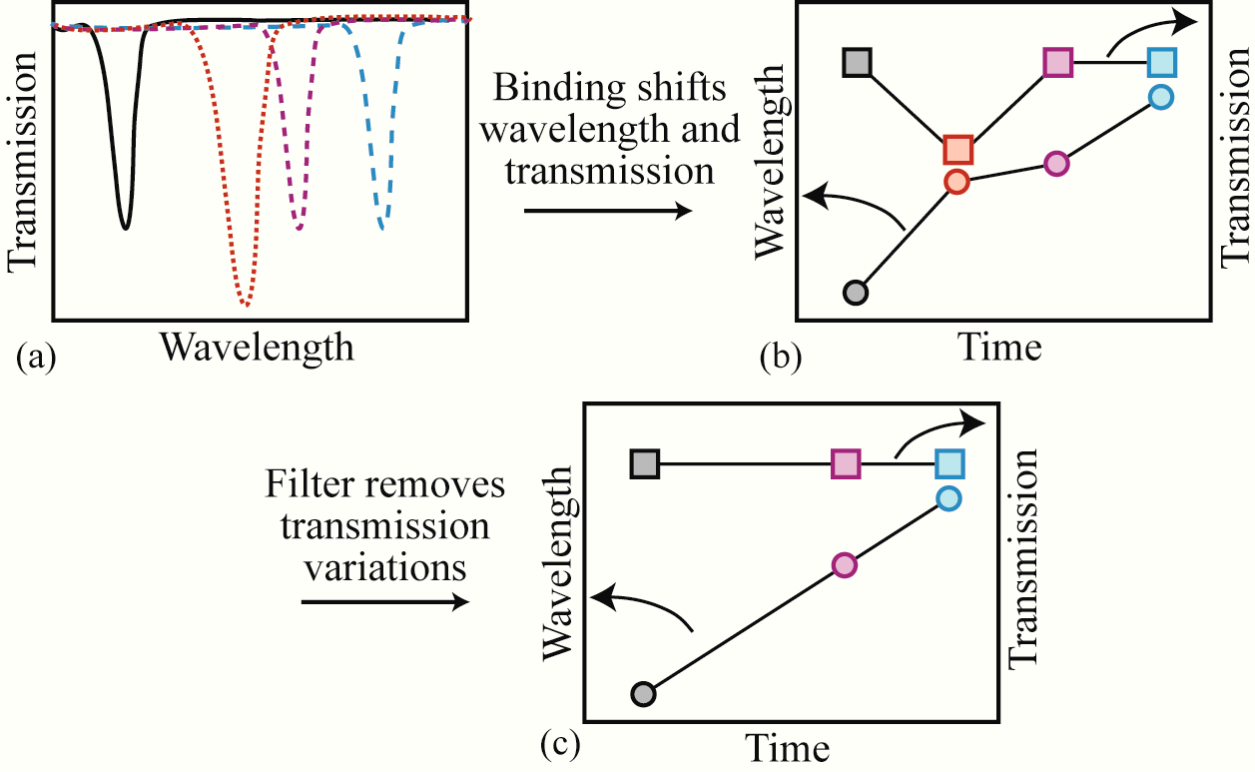
Image of the data analysis path. (**a**) The resonant wavelength red-shifts as proteins bind on the surface. Additionally, the transmission changes due to coupling instabilities; (**b**) if the four results in part (a) are re-plotted on a dual y-axis graph, the relationship between the wavelength and the transmission becomes apparent. Additionally, it becomes clear that this relationship can convolute the data analysis; (**c**) by applying a filter to remove coupling variations, the relationship between wavelength and time becomes evident.

The two common approaches for performing resonant cavity detection are: (1) tracking the resonant cavity wavelength (*x*-axis position) and (2) tracking the transmission or coupling (*y*-axis position). It is extremely difficult to track both in parallel using the standard resonant cavity-testing set-up, which relies on an external oscilloscope (wavelength tracking) or a power meter (transmission). However, when acquiring data at high acquisition speeds and high input powers, changes in coupling can give rise to small wavelength changes, leading to a high error rate or background noise. To solve this problem, we replaced the external oscilloscope with an integrated PCI high-speed digitizer/oscilloscope card. Using this integrated system in combination with a custom LabView data acquisition code, we are able to record both the wavelength and the transmission simultaneously. We can then apply a post-processing filter and remove all wavelength shift data where the transmission fluctuated by more than a predetermined amount. An image graphically explaining this analysis process is shown in Figure 3.

Once the variation in coupling is removed from the data, the results are further analyzed by plotting the normalized resonant wavelength shift (Δλ), or λ_i_ − λ_o_, where λ_i_ is the *i*th resonant wavelength and λ_o_ is the initial resonant wavelength value. From these results, the instantaneous shift (δλ) is determined (λ_i_ − λ_i−1_) and plotted as a histogram. However, this histogram includes instantaneous shifts for both the binding events and the noise. Therefore, this analysis is performed for both the detection experiments and for the control noise measurements. The histogram of the noise is fit to a Gaussian, and the 3σ value is determined. This decision represents our focus on optimizing the device and data analysis for minimum false-positives. However, it also means that some of our binding events will be excluded from analysis because they will fall within the 3σ value. Finally, the instantaneous shift from the noise is removed from the detection results, leaving only the signal from the protein binding.

## 3. Results and Discussion

### 3.1. Device Selection

It is well-known that the resonant shift is dependent on the refractive index contrast and the mode volume. Recently, it was also demonstrated that the evanescent field extension into the environment plays a significant role. In the present series of measurements using two different device geometries, we also observe consistency issues, both in the resonant frequency shift due to binding, as well as the background noise due to thermal effects. For example, in toroidal cavities, it is possible to achieve an athermal condition by balancing the positive thermo-optic coefficient of silica and the negative thermo-optic coefficient of water. However, due to the difference in optical mode profile and overlap with the environment, this condition is not possible with spheres. This change gives rise to a completely different background noise profile and thermal drift behavior.

Therefore, while the toroidal data or sphere data is self-consistent, comparing between device geometries is problematic. Investigations are currently underway to elucidate all of the different contributors, both geometry-specific parameters as well as others. However, at this time, it is not appropriate to directly compare results between devices given the previous work in the field, which has shown these numerous geometry-specific dependencies. As such, to facilitate the discussion of the results, we are focusing on the toroid data for the present work.

### 3.2. Wavelength Shift and Background Noise

**Figure 4:**
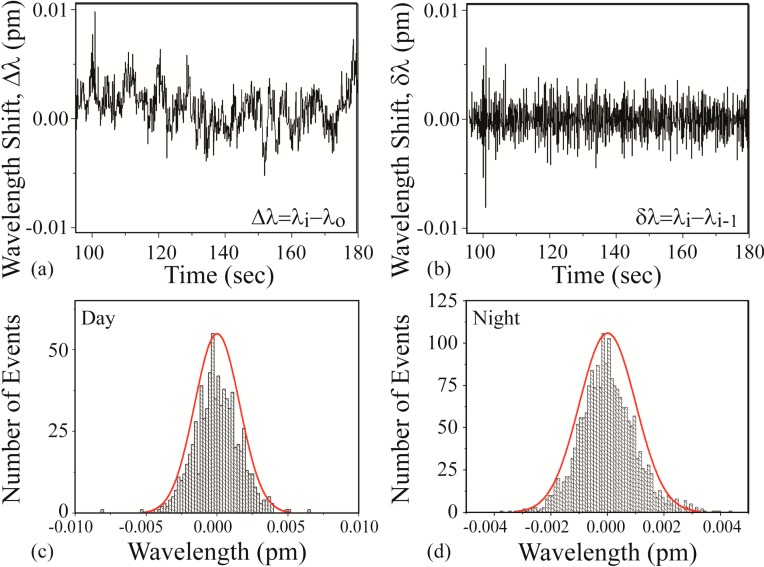
Noise measurements. (**a**) Normalized resonant wavelength shift (Δλ) measured during the day; (**b**) instantaneous resonant wavelength shift (δλ) of part (a); (**c**) histogram of the values in part (b); (**d**) histogram of values of an instantaneous resonant wavelength shift (δλ) measured at night.

At the start of every experiment, a background noise measurement is performed, and representative instantaneous noise histograms are shown in Figure 4. It is important to note that researchers in this field frequently interchange the names instantaneous shift (δλ) and the normalized shift (Δλ) with the term resonant wavelength shift. However, as can be seen in Figure 4a,b, these are distinct values and represent the results from different calculations. The results in Figure 4b can be more clearly understood if plotted as a histogram, where the y-axis represents the number of times that a single δλ value occurs (Figure 4c). As expected from a noise data set, it is very symmetric and exhibits a Gaussian distribution around the δλ = 0 point. For all sensing measurements, we set the noise threshold at 3 standard deviations (3σ) of the mean of this histogram. 3σ accounts for 99.7% of the noise. This experimentally-derived value is used to calculate the signal:noise (SNR) for each detection result. This thresholding approach works well, assuming a Gaussian noise distribution.

However, because detection measurements can be performed either during the day or at night, it is useful to compare the noise during these two times. The data in Figure 4a–c are taken during the day whereas the data in Figure 4d are taken at night.

As can be observed, the noise during the day is significantly higher (nearly twice) than the noise in the evening. Given this dependence, the noise is measured for each experiment uniquely. While this noise could be attributed to a range of noise sources, given the reproducibility of the data and the uniformity of the noise, we believe it is due to the air conditioning, which is reduced in the evening due to California energy saving regulations. At the time of these experiments, both lasers and the accompanying detectors were under one year old; therefore, noise due to the laser or the grating degradation was minimal. These findings inspired several subsequent investigations into the noise limitations in these types of experiments and experimental set-ups and alternative methods of analyzing the detection signal to circumvent these limitations [44,58,59].

### 3.3. Detection Measurements

**Figure 5:**
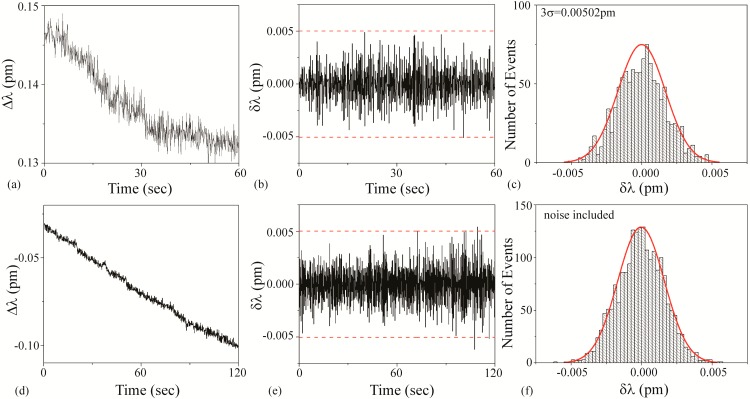
Cat solution: (**a**–**c**) background and (**d**–**f**) detection experiments. (**a**) Background wavelength shift (Δλ) results; (**b**) background instantaneous wavelength shift (δλ) results calculated from part (a) data. The dashed line is the 3σ noise level determined in part (c); (**c**) histogram of background δλ results calculated from part (b) data. Based on these results, the 3σ noise is 0.00502 pm; (**d**) wavelength shift (Δλ) upon injection of Cat solution; (**e**) instantaneous wavelength shift (δλ) results calculated from part (d) data. The dashed line is the 3σ noise level determined from the histogram shown in part (c); (**f**) histogram of δλ results shown in part (e). The background noise has not been subtracted from the histogram.

**Figure 6:**
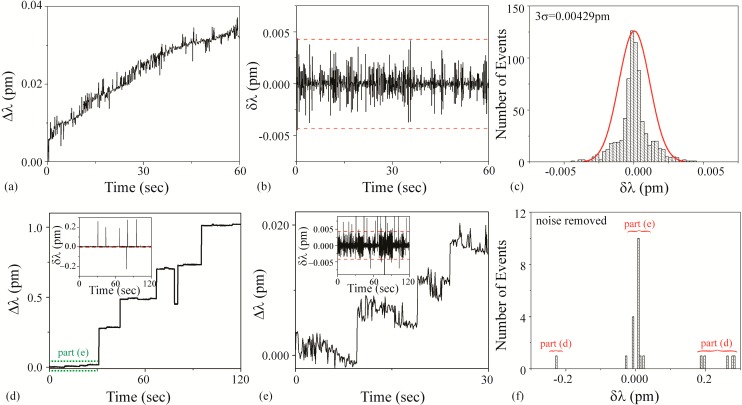
Cow solution: (**a**–**c**) background and (**d**–**f**) detection experiments. (**a**) Background wavelength shift (Δλ) results; (**b**) background instantaneous wavelength shift (δλ) results calculated from part (a) data. The dashed line is the 3σ noise level determined in part (c); (**c**) histogram of background δλ results calculated from part (b) data. Based on these results, the 3σ noise is 0.00429 pm; (**d**) wavelength shift (Δλ) upon injection of Cow solution. Inset: Instantaneous wavelength shift (δλ) results calculated from this data. The dashed line is the 3σ noise level determined from the histogram shown in part (c); (**e**) wavelength shift (Δλ) during first 30 s upon injection of Cow solution (region indicated in green in part (d)), highlighting small shifts. Inset: Instantaneous wavelength shift (δλ) results calculated from this data. The dashed line is the 3σ noise level determined from the histogram shown in part (c); (**f**) histogram of δλ results shown in part (d). The background noise has been subtracted from the histogram. Both the small and the large shifts are evident.

**Figure 7:**
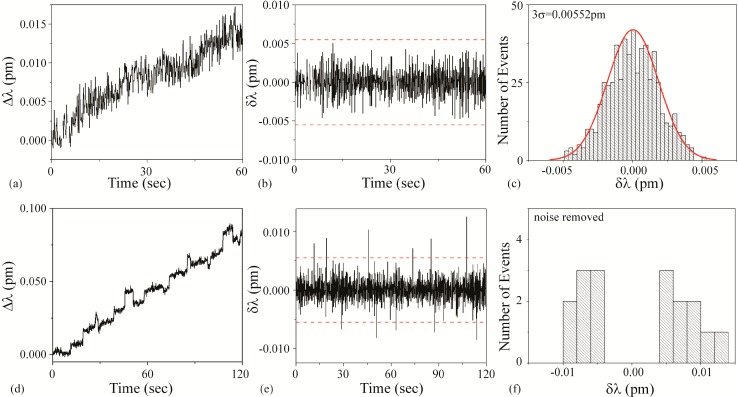
Dog solution: (**a**–**c**) background and (**d**–**f**) detection experiments. (**a**) Background wavelength shift (Δλ) results; (**b**) background instantaneous wavelength shift (δλ) results calculated from part (a) data. The dashed line is the 3σ noise level determined in part (c); (**c**) histogram of background δλ results calculated from part (b) data. Based on these results, the 3σ noise is 0.00552 pm; (**d**) wavelength shift (Δλ) upon injection of Dog solution; (**e**) instantaneous wavelength shift (δλ) results calculated from part (d) data. The dashed line is the 3σ noise level determined from the histogram shown in part (c); (**f**) histogram of δλ results shown in part (e). The background noise has been subtracted from the histogram.

**Figure 8:**
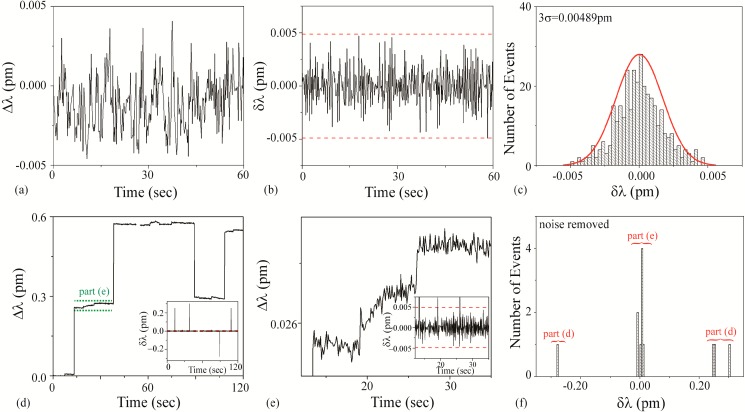
Pig solution: (**a**–**c**) background and (**d**–**f**) detection experiments. (**a**) Background wavelength shift (Δλ) results; (**b**) background instantaneous wavelength shift (δλ) results calculated from part (a) data. The dashed line is the 3σ noise level determined in part (c); (**c**) Histogram of background δλ results calculated from part (b) data. Based on these results, the 3σ noise is 0.00489 pm; (**d**) wavelength shift (Δλ) upon injection of Pig solution. Inset: Instantaneous wavelength shift (δλ) results calculated from this data. The dashed line is the 3σ noise level determined from the histogram shown in part (c); (**e**) wavelength shift (Δλ) upon injection of Pig solution (region indicated in green in part (d)), highlighting small shifts. Inset: Instantaneous wavelength shift (δλ) results calculated from this data. The dashed line is the 3σ noise level determined from the histogram shown in part (c); (**f**) histogram of δλ results shown in part (d). The background noise has been subtracted from the histogram. Both the small and the large shifts are evident.

Representative detection results and the resulting histograms from all four unknown solutions are shown in Figure 5, Figure 6, Figure 7 and Figure 8. Each measurement is shown with the noise data set, and the results will be discussed individually.

#### 3.3.1. Cat Solution

Figure 5a–c is the background noise measurements. Based on a fit to the histogram shown in Figure 5c, it was determined that the 3σ noise is 0.00502 pm. This value is plotted as dashed lines on Figure 5b,e. Figure 5d–f is the detection results using an unfunctionalized cavity upon the introduction of the Cat solution. During these measurements, the Q and the input power did not change.

It is important to note that the background noise was not removed in these measurements, as the magnitude of the noise-based shift is similar to that of solution-induced shift. This relationship is clearly observed by comparing Figure 5c,f or looking at the dashed lines in Figure 5e.

As a result, the SNR is approximately 1. An SNR of 1 is conventionally defined in the radar community as an undetectable signal. In the present work, that would translate to a “blank” solution, or the buffer. Therefore, based on the previously defined decision tree (Figure 1), the Cat solution is buffer.

#### 3.3.2. Cow Solution

Figure 6a–c is the background noise measurements. Based on a fit to the histogram shown in Figure 6c, it was determined that the 3σ noise is 0.00429 pm. This value is plotted as dashed lines on Figure 6b,d inset, and 6e inset. Figure 6d–f are the detection results using an unfunctionalized cavity upon the introduction of the Cow solution. During the injection, the Q and the transmitted power steadily decreased, most likely due to the agglomeration of the microparticles on the taper and the microcavity. Additionally, an increase in scattering at the surface of the cavity was observed during the experiments with a top view optical microscope and camera. This power and Q decrease limited the duration of experiments using these solutions.

As can be observed in Figure 6, the results from the Cow solution demonstrate two distinctly different shifts: a small shift (~0.007 pm) and a large shift (~0.2 pm). We concluded that the small shift is due to free streptavidin in the solution and the large shift is due to the SA-functionalized PS bead complex. This bi-magnitude shift was un-intended in the initial experimental design; however, it did reveal the ability of the method to detect impurities in a solution. This effect was further studied in subsequent work, looking into both binding rates and mass transport related effects [60]. Because of the dual signal, two different signal:noise calculations should be performed. Based on the raw data, for the large bead-streptavidin complex, the SNR is ~47 and for the streptavidin, the SNR is ~2.

#### 3.3.3. Dog Solution

In initial detection experiments characterizing Dog solution with unfunctionalized devices, shifts of approximately 0.01 pm are observed. This magnitude could correspond to either BSA or streptavidin. Therefore, to differentiate between BSA and streptavidin, the Dog solution is tested with a biotin-functionalized device. During the injection and detection measurements, the Q and transmitted power did not change.

Figure 7a–c is the background noise measurements using a functionalized device. Based on a fit to the histogram shown in Figure 7c, it was determined that the 3σ noise is 0.00552 pm. This value is plotted as dashed lines on Figure 7b,e. Figure 7d–f is the detection results.

As can be observed in Figure 7, the small shifts, which are indicative of small protein binding events, are still present. Based on the decision tree outlined in Figure 1, we conclude that the Dog solution contains streptavidin. Based on the raw data, the SNR is ~2.

#### 3.3.4. Pig Solution

Figure 8a–c is the background noise measurements. Based on a fit to the histogram shown in Figure 8c, it was determined that the 3σ noise is 0.00489 pm. This value is plotted as dashed lines on Figure 8b,d inset, and 8e inset. Figure 8d–f are the detection results using an unfunctionalized cavity upon the introduction of the Pig solution. During the injection, the Q and the transmitted power steadily decreased and an increase in scattering on the cavity surface were observed. These effects were most likely due to the agglomeration of the microparticles on the taper and the microcavity. This power and Q decrease limited the duration of experiments using these solutions.

As can be observed in Figure 8, the results from the Pig solution demonstrate two distinctly different shifts: a small shift (~0.007 pm) and a large shift (~0.2 pm). We concluded that the small shift is due to the presence of free streptavidin in the solution and the large shift is due to the SA-functionalized PS bead complex. This bi-magnitude shift was un-intended in the initial experimental design; however, it did reveal the ability of the method to detect impurities in a solution. Because of the dual signal, two different signal:noise calculations should be performed. Based on the raw data, for the large bead-streptavidin complex, the SNR is ~40 and for the streptavidin, the SNR is ~1.5. It is important to note that because the Pig and the Cow solutions are both the PS-bead complex, by default, not all of the known solutions (buffer, BSA, SA, and PS bead complex) will be in the set of unknowns.

### 3.4. Summary of Measurements

Table 1 summarizes all of the results and experimental observations. It also includes the prediction based on the experimental results and the actual solution from the sealed key. It is notable that the experimental results were able to accurately predict all four of the solutions. Based purely on probability, there is a 25% chance of identifying any single solution correctly. Given the limited information we were provided, the probability of identifying all four correctly is 0.4%. Therefore, this level of accuracy is truly based on the sensor’s performance.

**Table 1:** Comparison of predicted and actual (known) solutions.

Solution	Buffer	BSA	SA	SA-PS Bead	Cat	Dog	Cow	Pig
**Prediction**					Buffer	SA	SA-PS bead	SA-PS bead
**Actual**					Buffer	SA	SA-PS bead	SA-PS bead
**General observations and/or comments which led to this conclusion**	No shifts over noise level; no change in cavity Q	δλ ~0.01 pm; minimal change in Q; minimal change in coupled power	δλ ~0.01 pm; minimal change in Q; minimal change in coupled power	δλ > 0.2 pm and smaller shifts; power and Q decrease; scattering observed	No shifts over noise level; no change in cavity Q	δλ ~0.01 pm	δλ > 0.2 pm and smaller shifts; power and Q decrease over numerous trials	δλ > 0.2 pm and smaller shifts; power and Q decrease over numerous trials

### 3.5. Circulating Power and Thermal Effects

As was mentioned in the procedures, the present series of experiments used very high input powers. This approach will give rise to very high circulating power or circulating intensity inside the optical cavity. Specifically, the circulating power can be analytically calculated as:

$$P_{circ}=\frac{\lambda Q}{\pi^{2}n_{eff}R_{meff}}\frac{K}{{\left(1+K\right)}^{2}}$$

where λ is the resonant wavelength, n_eff_ is the effective refractive index, Q is the intrinsic cavity quality factor, K is the coupling constant and R_meff_ is the effective radius of the mode. R_meff_ is related to the cavity major and minor diameters. Due to material absorption, temperature gradients are created [61]. Therefore, this circulating power can be observed as a change in the resonant wavelength according to:

$$\text{Δ}\lambda =(\frac{\lambda }{n_{eff}})(\frac{dn}{dT})(\text{Δ}T)+\lambda \varepsilon \text{Δ}T$$

where dn/dT is the effective thermo-optic coefficient, ΔT is the temperature change, and ε is the thermal expansion of the material. Typically, in dielectric systems, it is assumed that the index change dominates this equation. However, in certain situations, it has been shown that it is possible to remove the index-generated effect by balancing the positive dn/dT of silica with a negative dn/dT material, creating a net-zero dn/dT [62,63,64,65,66]. In sensing applications, this null shift is particularly desirable, as it will reduce the background signal. As can be seen throughout all of the noise measurements, we strived to achieve this neutrality condition by manipulating the optical field overlap between the silica device (positive dn/dT) and water (negative dn/dT). Due to subtle variations in device geometry, we observed a range of behaviors, including blue and red shifts as well as the ideal neutrality condition. However, the magnitude of these shifts is minor when compared to the shifts that would be observed if the device was operating in air, which has a positive dn/dT.

## 4. Conclusions/Outlook

In the present work, we have successfully performed a double-blind experiment verifying the ability of optical resonant cavities to detect and to identify single molecules based on resonant wavelength shifts. Additionally, we developed several new data analysis methods, enabling sub-linewidth detection limits without employing complex experimental techniques, such as heterodyning [42,67] or back-scatter [6]. These measurements have already inspired numerous subsequent research efforts into signal optimization and noise analysis as well as surface chemistry engineering [54,58,59]. Therefore, in addition to impacting the immediate field of resonant cavity biosensing, these findings have already and will continue to advance the broader field of biological and chemical sensing.
